# Supplementary material for: The Structural Flexibility of PR-10 Food Allergens
Source: Int J Mol Sci. 2022 Jul 26;23(15):8252. doi: 10.3390/ijms23158252 (PMC9330593; doi:10.3390/ijms23158252)
Supplement: Supplementary file 1 [file ijms-23-08252-s001.zip › ijms-1832702-supplementary.pdf]

The structural flexibility of PR-10 food allergens

Sebastian Führer, Jana Unterhauser, Ricarda Zeindl, Reiner Eidelpes, Monica L. Fernández-Quintero, Klaus R. Liedl and Martin Tollinger

```
Bet v 1.0101 GVFNYETETT SVIPAARLFK AFILDGDNLF PKVAPQAISS VENIEGNGGP GTIKKISFPE GFPFKYVKDR VDEVDHTNFK 80
Mal d 1.0101 GVYTFENEFT SEIPPSRLFK AFVLDADNLI PKIAPQAIKQ AEILEGNGGP GTIKKITFGE GSQYGYVKHR IDSIDEASYS 80
Act c 8.0101 GVVTYDMEIP SKVPPVKLYK AFILDGDTLV PKVLPHAIKC VKILEGDGCA GTIKEVTFGE GSHHKCVKQR VDAIDKDNLT 80
Act d 8.0101 GAITYDMEIP SSISAEKMFK AFVLDGDTII PKALPHAITG VQTLEGDGGV GTIKLTTFGE GSVHKSVKHR IDGLDKENFT 80
Pru d 1.0101 GVFTYESEFT SEIPPPRLFK AFVLDADNLV PKIAPQAIKH SEILEGDGGP GTIKKITFGE GSQYGYVKHK IDSIDKENHS 80
Pru p 1.0101 GVFTYESEFT SEIPPPRLFK AFVLDADNLV PKIAPQAIKH SEILEGDGGP GTIKKITFGE GSQYGYVKHK IDSIDKENHS 80
Pru p 1.0201 GVFTYSDEST SVIPPPRLFK ALVLEADTLI PKIAPQSVKS AEIVEGDGGV GTIKKISFGE GSHYSYVKHR IDGLDKDNFV 80
Pru p 1.0301 GVFTYETEFT SVIPPEKLFK AFILDADNLI PKIAPTAVKD TEILEGDGGV GTIKKVTFGE GSQYGYVKHR IDGIDKDNLT 80
Ara h 8.0101 GVFTFEDEIT STVPPAKLYN AM-KDADSIT PKIID-DVKS VEIVEGNGGP GTIKKLTIVE DGETKFILHK VESIDEANYA 78
Cor a 1.0401 GVFCYEDEAT SVIPPARLFK SFVLDADNLI PKVAPQHFTS AENLEGNGGP GTIKKITFAE GNEFKYMKHK VEEIDHANFK 80
Cor a 1.0402 GVFSYEDEAT SVIPPARLFK SFVLDADNLI PKVAPQHFTG AENLEGNGGP GTIKKITFAE GSEFKYMKHK VEEIDHANFK 80
Cor a 1.0403 GVFCYEDEAT SVIPPARLFK SFVLDADNLI PKVAPQHFTG AENLEGNGGP GTIKKITFAE GSEFKYMKHK VEEIDHANFK 80
Cor a 1.0404 GVFSYEDEAT SVIPPARLFK SFVLDADNLI PKVAPQHFTS AENLEGNGGP GTIKKITFAE GNEFKYMKHK VEEIDHANFK 80
             *.  :. *   * :   :::: ::  :.*.:  **     ..   : :**:*   ****  :: * .     : .: :: :*. .

Bet v 1.0101 YNYSVIEGGP IGDTLEKISN EIKIVATPD- GGSILKISNK YHTKGDHEVK AEQVKASKEM GETLLRAVES YLLAHSDAYN 159
Mal d 1.0101 YSYTLIEGDA LTDTIEKISY ETKLVACGS- -GSTIKSISH YHTKGNIEIK EEHVKVGKEK AHGLFKLIES YLKDHPDAYN 158
Act c 8.0101 YSYTIIEGDV LAEKFESISY HIKIVACPD- GGSICKNRSI YTTKGDCKVS EEEIKLGKEK AAEIFKALEA YLLANPDYC- 158
Act d 8.0101 YSYSIIEGGA L-DVFESISY HIKIVATPD- GGCICKNRSI YTPKCDAQVS EEEIKAGKER ASGIFKKVEA YLLANPDC-- 156
Pru d 1.0101 YSYTLIEGDA LGDNLEKISY ETKLVASPS- GGSIIKSISH YHTKGDVEIK EEHVKAGKEK ASNLFKLIET YLKGHPDAYN 159
Pru p 1.0101 YSYTLIEGDA LGDNLEKISY ETKLVASPS- GGSIIKSTSH YHTKGDVEIK EEHVKAGKEK ASNLFKLIET YLKGHPDAYN 159
Pru p 1.0201 YNYTLVEGDA LSDKVEKITY EIKLVASAD- GGSIIKSTSN YHTKGDVEIK EEDVKAGKEK ATGLFKLIEN YLVANPDAYN 159
Pru p 1.0301 YSYTLIEGDA LSDVIEKIVY DIKLVASPN- GGSIVKTISH YHTKGDVEIK EEQVKAGKEK AAGLFKLVEG YLLANPDAYN 159
Ara h 8.0101 YNYSVVGGVA LPPTAEKITF ETKLVEGPN- GGSIGKLTLK YHTKGDAKPD EEELKKGKAK GEGLFRAIEG YVLANPTQY- 156
Cor a 1.0401 YCYSIIEGGP LGHTLEKISY EIKMAAAPHG GGSILKITSK YHTKGNASIN EEEIKAGKEK AAGLFKAVEA YLLAHPDAYC 160
Cor a 1.0402 YCYSIIEGGP LGHTLEKISY EIKMAAAPHG GGSILKITSK YHTKGNASIS EEEIKAGKEK AAGLFKAVEA YLLAHPDTYC 160
Cor a 1.0403 YCYSIIEGGP LGHTLEKISY EIKMAAAPHG GGSILKITSK YHTKGNASIS EEEIKAGKEK AAGLFKAVEA YLLAHPDTYC 160
Cor a 1.0404 YCYSIIEGGP LGHTLEKIPY EIKMAAAPHG GGSILKITSK YHTKGNASIN EEEIKAGKEK AAGLFKAVEA YLLAHPDAYC 160
             * *::: *   :    *.*   . *:.       *.  *     *  * : . .  *.:* .*   .  ::: :*  *:  :
```

Supporting Figure S1. Sequence alignment of PR-10 allergens. Identical amino acid residues are labeled with asterisks, conserved residues with colons and semiconserved residues with dots. Positions of amino acid residues that are not present in a specific allergen are indicated by a dash.

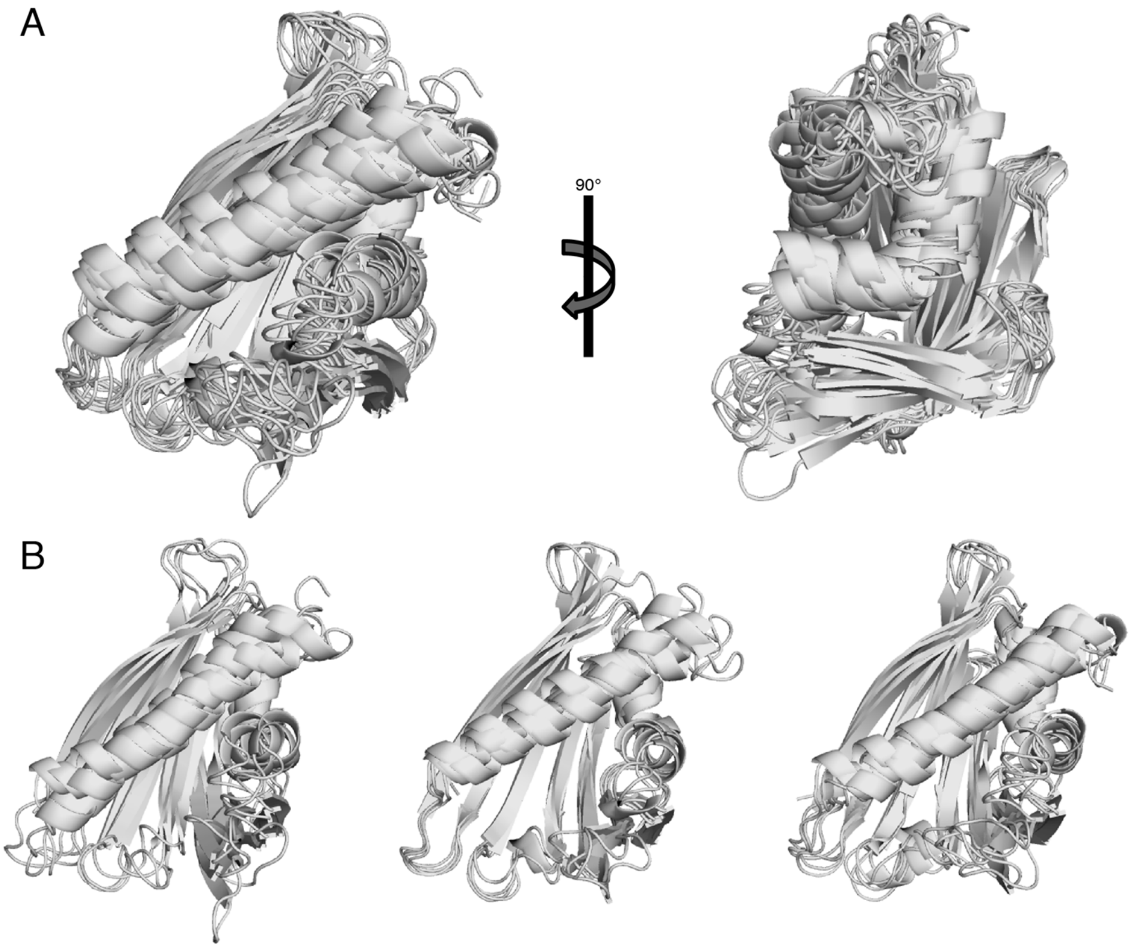

Supporting Figure S2. Structural comparison of the thirteen PR-10 allergens in this study. (**A**) Overlay of all structures. (**B**) Overlay of Cor a 1.0101, Cor a 1.0402, Cor a 1.0403, Cor a 1.0404 (left) compared to Pru p 1.0101, Pru p 1.0201, Pru p 1.0301, Pru d 1.0101 (middle) and Bet v 1.0101, Mal d 1.0101, Ara h 8.0101, Act c 8.0101, Act d 8.0101 (right).

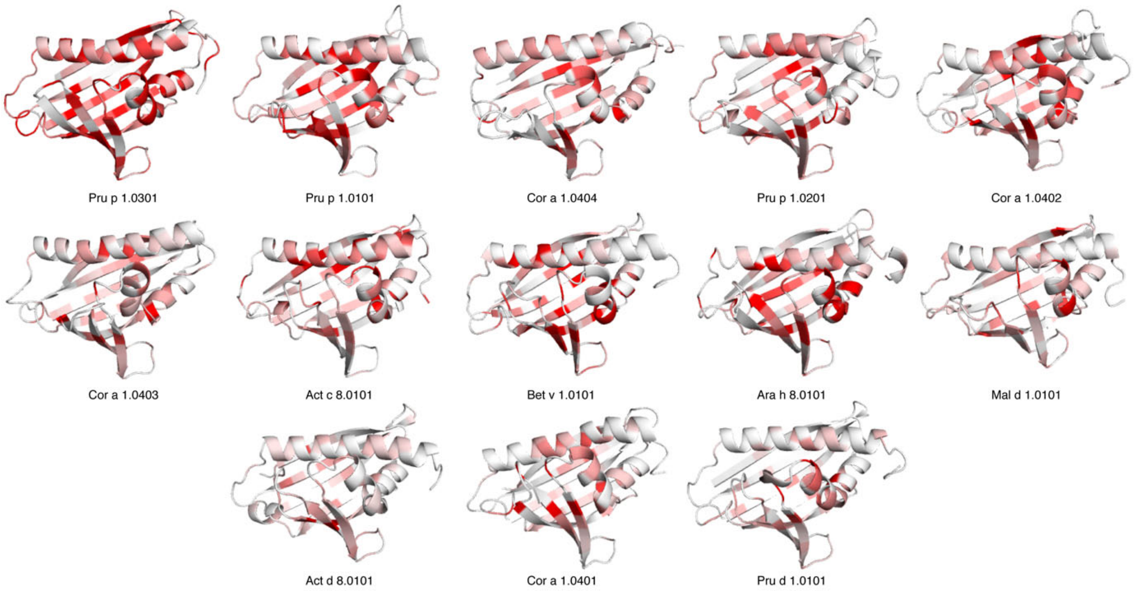

Supporting Figure S3. Structural flexibility of the thirteen PR-10 allergens. Relaxation dispersion amplitudes ($\Delta R_{2,eff}$ values) exceeding 1 s^{-1} at 700 MHz are plotted on the protein backbones, using a color gradient from rigid (white) to more flexible (red). The same color gradient as in Figure 3 was used for all proteins.

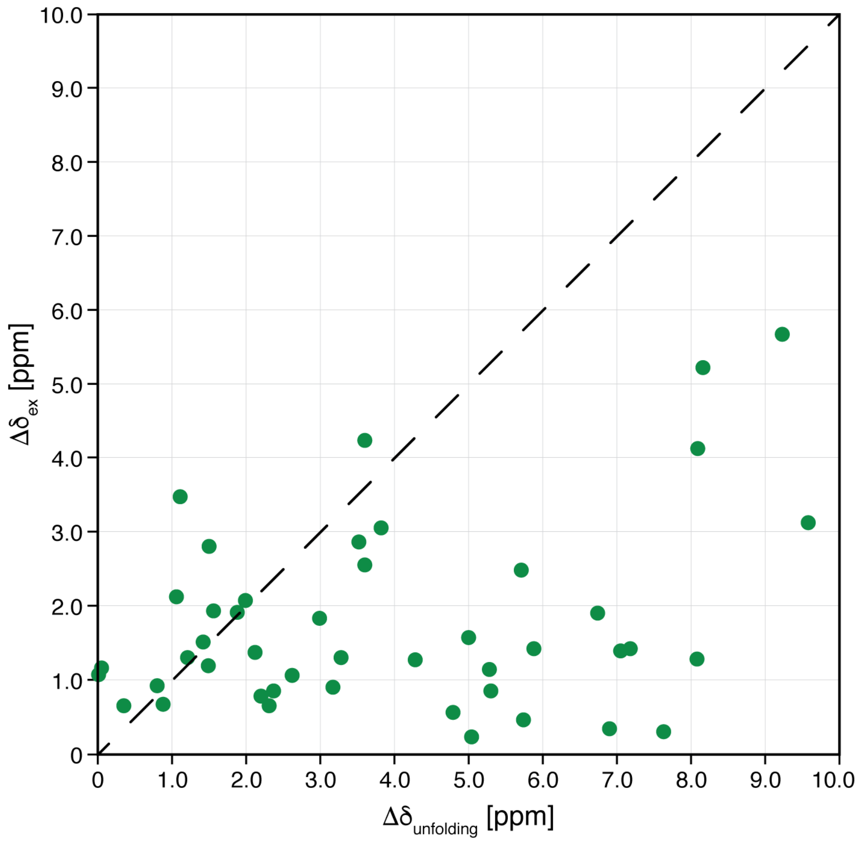

Supporting Figure S4. Comparison of ^{15}N chemical shift differences, $\Delta\delta_{ex}$, obtained from the backbone amide ^{15}N relaxation dispersion data of Ara h 8.0101 with ^{15}N secondary chemical shifts, $\Delta\delta_{unfolding}$, as expected for complete unfolding of the protein backbone. Values of $\Delta\delta_{unfolding}$ were calculated as the difference between observed ^{15}N chemical shifts and predicted ^{15}N chemical shifts for a random coil (Tamiola K, Acar B, Mulder FAA, *J Am Chem Soc* 2010 (132), 18000-18003. doi: 10.1021/ja105656t). The dashed, drawn with a slope of 1.0, line indicates the ^{15}N chemical shift differences, $\Delta\delta_{ex}$, that are expected for complete unfolding of the backbone to a random coil.

Helices		Sheet	
Ala16	0.30 ± 0.16	Lys37	1.51 ± 0.34
Lys 17	0.46 ± 0.06	Ser38	5.22 ± 0.51
Asn20	0.85 ± 0.07	Val39	2.80 ± 0.25
Ala21	1.83 ± 0.14	Ile41	1.30 ± 0.23
Met22	0.78 ± 0.06	Glu43	1.42 ± 0.27
Lys23	1.39 ± 0.11	Gly47	2.55 ± 0.29
Asp24	0.92 ± 0.07	Thr50	0.23 ± 0.10
Ala25	1.06 ± 0.07	Lys53	1.16 ± 0.25
Ser27	1.57 ± 0.13	Thr55	1.42 ± 0.14
Lys31	2.48 ± 0.21	Ile56	2.07 ± 0.22
Ile33	0.65 ± 0.07	Leu66	3.47 ± 0.24
Asp123	0.90 ± 0.14	His67	3.05 ± 0.16
Glu128	1.37 ± 0.10	Asp73	1.93 ± 0.17
Gly134	1.19 ± 0.08	Glu74	1.14 ± 0.12
Lys137	1.07 ± 0.08	Val84	3.12 ± 1.08
Gly138	0.65 ± 0.19	Val87	1.90 ± 0.26
Glu139	0.85 ± 0.12	Ala88	5.67 ± 0.81
Ile145	0.67 ± 0.08	Leu89	1.30 ± 0.17
Glu146	0.56 ± 0.07	Thr97	4.12 ± 0.90
Tyr156	0.25 ± 0.16	Phe98	4.23 ± 1.04
		Glu99	1.91 ± 0.16
		Thr100	1.27 ± 0.22
		Lys101	2.86 ± 0.30
		Leu102	2.21 ± 0.18
		Thr115	0.34 ± 0.18
		Tyr118	1.28 ± 0.36

Supporting Table S1. Residue-specific ^{15}N chemical shift differences, $\Delta\delta_{ex}$, between major and minor states, obtained from sub-global fits of the backbone amide ^{15}N relaxation dispersion data for amino acid residues in the three α-helices and loop L2 (left) and in the β-sheet and all other loops (right). Data are shown for Ara h 8.0101. For amino acid residues highlighted in blue and orange the experimental relaxation dispersion data are shown in Figure 4.
